# Human ocular dirofilariasis in Poland

**DOI:** 10.1186/s12348-024-00401-5

**Published:** 2024-07-10

**Authors:** Piotr J. Gaca, Rafal Nowak, Robert Rejdak, Magdalena Rejdak, Maja Nowak, Beata Flis, Mohammad Javed Ali

**Affiliations:** 1 Chair and Department of General and Paediatric Ophthalmology, Medical University of Lublin, Lublin, Poland; 2 Eye Department, Jozef Strus City Hospital, Poznan, Poland; 3 Department of Ophthalmology, University Hospital Zurich, Zurich, Switzerland; 4 Govindram Seksaria Institute of Dacryology, L.V. Prasad Eye Institute, Hyderabad, India

## Introduction

Human dirofilariasis is a zoonotic disease caused by filarial parasites belonging to the Onchocercidae family. Filariae have no free-living stages, and they require a definitive host and an intermediate host in their lifecycle. Domestic and wild canids constitute the main reservoir for *Dirofilaria repens. This parasite is* transmitted by mosquitoes. In Europe, the primary vectors are the *Culex pipiens* and *Aedes albopictus* mosquitoes. Nevertheless, it has recently been suggested that other flying insects, such as black flies from the *Simulium turgaicum complex* and *Culicoides paolae* biting midges, could also play a role [1–3]. Humans are accidental hosts. In recent years, the prevalence of *D. repens* infestation has increased in Europe, with the majority of cases found in Mediterranean countries (southern Italy, southern France, and Greece), Ukraine, the Russian Federation and Belarus [4–7]. In infected humans, *D. repens* usually migrates subcutaneously, causing mild and unrecognized symptoms, and rarely reaches ocular tissues or other organs [4, 8]. The current paper reports the presentation and management of an extensive subconjunctival infestation caused by *D. repens* from Poznan, Poland.

The study adhered to the tenets of the Declaration of Helsinki.

## Case report

**Fig. 1:**
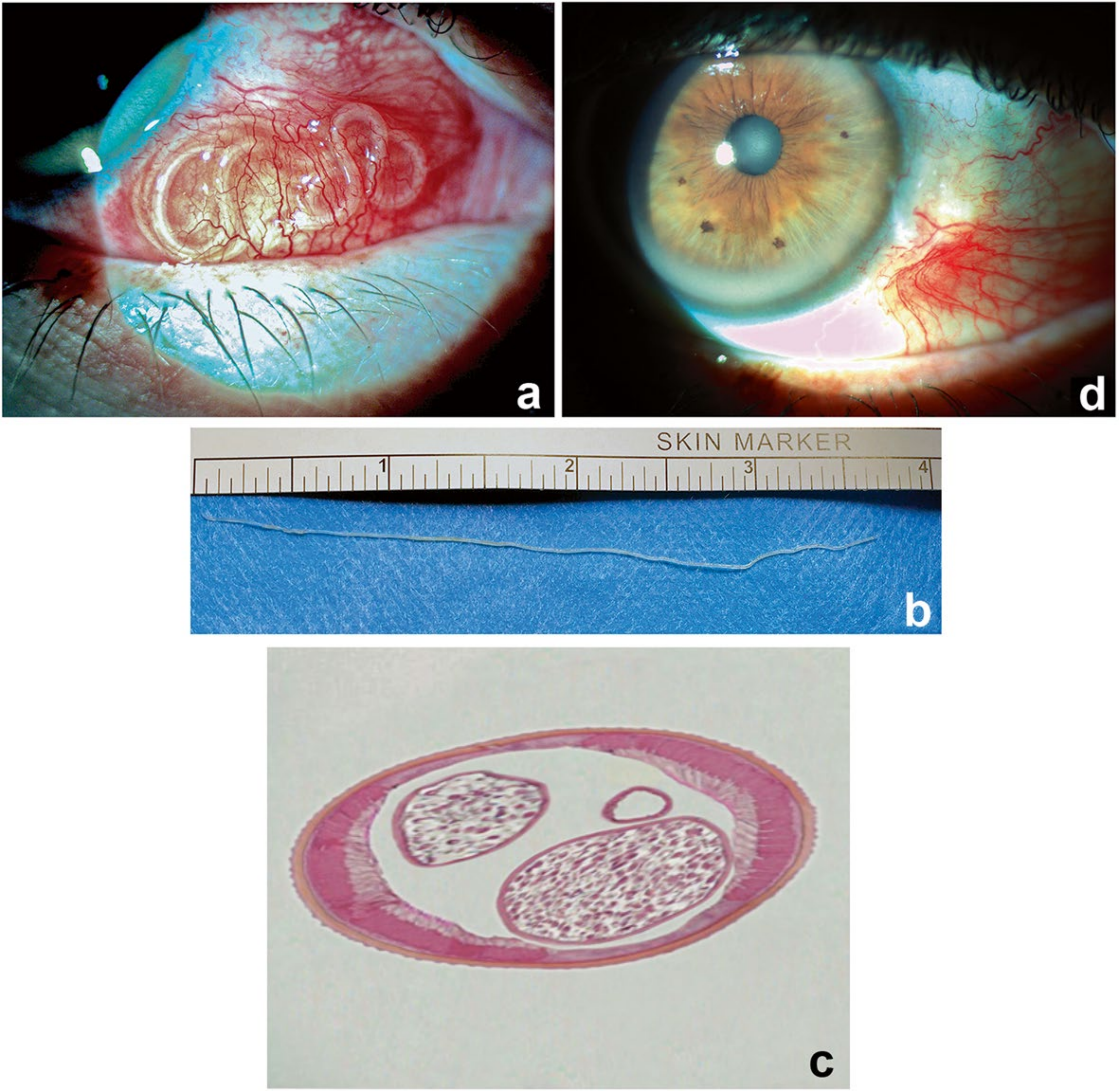
A subconjunctival greyish white nematode visible in the nasal aspect of the right eye (**a**). External image demonstrating the retrieval of the intact worm (**b**). Haematoxylin–eosin-stained transverse section of the worm (**c**). Postoperative image showing good surface healing with resolution of the conjunctival congestion (**d**)

In June 2023, a 63-year-old male farmer presented with complaints of redness, foreign body sensation, itching and pain in the right eye for one week. His best-corrected visual acuity (BCVA) was 20/20 in both eyes. An external inspection at presentation revealed conjunctival congestion and a nodule-like lesion on the nasal bulbar region of the right eye. Slit-lamp examination revealed a motile subconjunctival greyish-white to yellow unfolded nematode (Fig. 1a). Higher magnification revealed that the long, coiled, greyish-white worm was in constant motion (Video 1). The anterior and posterior segment examination of both eyes was otherwise unremarkable. The medical history was revised. The patient originated from western Poland. He had no dogs at his farm, and neither did his closest neighbours. The only country he had visited previously was England, several years before. There were no symptoms of cutaneous erythema, pruritus, or subcutaneous swelling. Investigations revealed the complete blood count to be unremarkable without any signs of eosinophilia. Other blood samples were sent to the hospital’s referral laboratory at the Clinic of Tropical and Parasitic Diseases in Poznan. An enzyme-linked immunosorbent assay (ELISA) for filarial antibodies was negative (ARG83067 Dirofilaria ELISA Kit, Arigo Biolaboratories Corp., Hsinchu City, Taiwan). The patient underwent surgery the next day under local anaesthesia. The conjunctiva was incised using Westcott scissors to expose the nematode. The worm was carefully removed in an intact manner with forceps, with its integrity being completely retained (Fig. 1b). The conjunctiva was closed with 7/0 resorbable sutures (Vicryl Rapide, Ethicon). Postoperatively, the patient was advised to use topical dexamethasone ointment for one week. The worm measured 10 cm in length (Fig. 1b). The worm was fixed in 4% buffered formaldehyde and sent to the Clinic of Tropical and Parasitic Diseases in Poznan for further tests. Because PCR examination was unavailable, histopathology was performed. Transverse sections stained with haematoxylin–eosin revealed the presence of longitudinal ridges on the cuticle and uteri that were indicative of female *D. repens* (Fig. 1c) [9, 10]. The patient was referred to the Clinic of Tropical and Parasitic Diseases in Poznan for further examination, where coprological and serological tests for parasitic diseases were negative. Microfilariae were not found in the direct blood smear. Currently, the patient undergoes regular follow-up for systemic monitoring. At the four-week follow-up visit at the ophthalmology clinic, slit lamp examination revealed a noninflamed and well-healed ocular surface (Fig. 1d).

## Discussion

Six species of *Dirofilaria*, *D. repens, D. immitis, D. striata, D. tenuis, D. ursi and D. spectans*, are known to affect humans. These species exhibit a complex lifecycle involving multiple developmental stages in both the definitive mammalian host (usually a dog or cat) and the intermediate host/vector (arthropods, mostly mosquitoes). Infested patients can exhibit cutaneous symptoms (itching, pruritus, movement below the skin, and subcutanous nodules) that can cause larva migrans syndrome.

When affecting the ocular region, the larva can migrate into the peri-, intra- or retro-ocular spaces and can involve the eyelids, lacrimal glands, subconjunctival space, cornea, sub-Tenon's space, anterior chamber, and vitreous body. *Dirofilaria* infestation has been reported to manifest as lacrimal gland swelling, episcleritis, uveitis, and glaucoma [4, 11–15]. In a group of 39 Austrian patients infected with *D. repens,* isolated involvement of the ocular tissues was noted in 23.1% (9/39) of patients [11]. An analysis of dirofilariasis in 102 patients in Ukraine revealed ocular or periocular locations in 52.9% of the sample [12]. Pupic-Bakrac et al. [14] reported 21 human infestations with female *D. repens*, and six (27.27%) of the patients had ocular dirofilariasis. A review by Kalogeropoulos et al. [11] revealed that most of the cases were subconjunctival in location (> 60% of all cases), followed by eyelid/orbital dirofilariasis (approximately 25%). Similarly, a study from Greece reported subconjunctival dirofilariasis (62.5%) to be common, followed by intravitreal dirofilariasis (25%) and orbital dirofilariasis (12.5%) [10]. One case report from India described the presence of dirofilaria in the anterior chamber of a patient [10].

A careful history of typical symptoms, contact with dogs, insect bites, and travel, especially to endemic areas of human dirofilaria infections, is helpful in diagnosis [4] While the clinical examination of the eye clearly establishes the presence of a worm and the initial diagnosis is based on morphological examination, the final detection of *D. repens* species should be based on species-specific PCR that amplifies a portion of the cytochrome oxidase subunit 1 (COI) gene [13]. In the event of PCR unavailability, histopathological examination may support the diagnostic process [8, 9].

The treatment of choice is surgical removal of the parasite. Larvae located within the eyelids, subconjunctivally, or in the corneas, sub-Tenon's spaces or the anterior chambers can usually be surgically removed without serious consequences. In rare cases of intravitreal dirofilariasis, patients undergo vitrectomy for the removal and subsequent identification of the nematode [4, 10, 14, 16]. Pharmacological treatment is used in rare cases where microfilariae are detected in the blood [4]. Due to the uncommon occurrence of this disease in humans, there are no consensus treatment guidelines. As dogs are the most important reservoir of the parasite, reducing the burden of their dirofilariasis should be a deliberate preventive measure to decrease the risk of human infection [4, 5, 17].

### Supplementary Information

**Supplementary Material 1.**

## Data Availability

No datasets were generated or analysed during the current study.
